# Adipokine secretion and lipolysis following gender-affirming treatment in transgender individuals

**DOI:** 10.1007/s40618-024-02323-4

**Published:** 2024-03-09

**Authors:** N. Subramanian, A. Wiik, E. Rullman, M. Melin, T. R. Lundberg, J. Flanagan, M. Holmberg, A. Dekanski, C. Dhejne, S. Arver, T. Gustafsson, J. Laurencikiene, D. P. Andersson

**Affiliations:** 1 Lipid Laboratory, Department of Medicine Huddinge (H7), Karolinska Institutet, C2:94, Karolinska University Hospital Huddinge, 141 86 Huddinge, Sweden; 2 Department of Laboratory Medicine, Division of Clinical Physiology, Karolinska Institutet, and Unit of Clinical Physiology, Karolinska University Hospital, Stockholm, Sweden; 3 Department of Cardiology, Heart and Vascular Center, Karolinska University Hospital, Stockholm, Sweden; 4 ANOVA, Andrology, Sexual Medicine and Transgender Medicine, Karolinska University Hospital, Stockholm, Sweden; 5 Department of Medicine Huddinge, Karolinska Institutet, Stockholm, Sweden; 6 Department of Endocrinology, Karolinska University Hospital Huddinge, Stockholm, Sweden

**Keywords:** Adipose tissue, Lipolysis, Transgender, Transmen, Transwomen

## Abstract

**Background:**

The organ-specific effects of gender-affirming sex hormone treatment (GAHT) in transgender women (TW) and transgender men (TM) are insufficiently explored. This study investigated the effects of GAHT on adipose tissue function.

**Methods:**

In a single-center interventional prospective study, 32 adults undergoing GAHT, 15 TW and 17 TM, were examined with anthropometry and abdominal subcutaneous adipose tissue biopsies obtained before initiation of treatment, 1 month after endogenous sex hormone inhibition and three and 11 months after initiated GAHT. Fat cell size, basal/stimulated lipolysis and cytokine secretion in adipose tissue were analyzed.

**Results:**

TW displayed an increase in complement component 3a and retinol-binding protein 4 (RBP4) secretion after sex hormone inhibition, which returned to baseline following estradiol treatment. No changes in lipolysis were seen in TW. TM showed downregulation of RBP4 after treatment, but no changes in basal lipolysis. In TM, the estrogen suppression led to higher noradrenaline stimulated (NA) lipolysis that was normalized following testosterone treatment. At 11 months, the ratio of NA/basal lipolysis was lower compared to baseline. There were no significant changes in fat cell size in either TW or TM.

**Conclusion:**

In TW, gonadal hormone suppression results in transient changes in cytokines and in TM there are some changes in NA-stimulated lipolysis following testosterone treatment. However, despite the known metabolic effects of sex hormones, the overall effects of GAHT on adipose tissue function are small and likely have limited clinical relevance, but larger studies with longer follow-up are needed to confirm these findings.

**Trial registration:**

ClinicalTrials.gov Identifier: NCT02518009, Retrospectively registered 7 August 2015.

**Supplementary Information:**

The online version contains supplementary material available at 10.1007/s40618-024-02323-4.

## Introduction

Gender dysphoria is a condition characterized by distress from an incongruence between a person’s gender identity and their sex recorded at birth. There has been an increase of individuals seeking gender-affirming treatment [1, 2]. Possible treatments include psychological counseling as well as gender-affirming hormonal treatment (GAHT) and surgical procedures aimed at aligning the body with the gender identity. The hormonal treatment consists of downregulation of endogenous gonadal hormones and gender-affirming hormonal treatment.

There is ample evidence that sex hormones have strong systemic and local metabolic and immunomodulatory effects [3–6]. The effects of gender-affirming hormone treatment on clinical outcomes such as cardiovascular disease [7–9], mortality [10], body composition [11], incretin levels and insulin sensitivity [12] have been studied previously. However, very few studies have addressed organ-specific (e.g., adipose-specific) metabolic effects at the cellular level. The pernicious phenotypes of adipose tissue, including enlargement of fat cells and altered release of fatty acids (although basal lipolysis is increased and stimulated lipolysis is decreased) and release of inflammatory factors are associated with insulin resistance [13–15], dyslipidemia [13], metabolic disease [16] and weight gain [17] over time. Furthermore, several adipose tissue phenotypes, such as fat cell size, level of lipolysis and secretion of cytokines show sex dimorphism [13, 17, 18] and in vitro studies have shown that both estrogen and testosterone reduce stimulated lipolysis [6, 19]. An accumulation of visceral/upper body fat often seen in males confers an increased cardio-metabolic risk. In addition, fat cell size, which is negatively associated with insulin sensitivity [13], is regulated differently in males and females. Lean females have larger subcutaneous fat cells than lean males on average. On the other hand, in individuals with a larger amount of adipose tissue, the opposite is seen [20]. Furthermore, one of the important adipose tissue anti-inflammatory cytokines, IL-10, is upregulated only in obese females, suggesting a possible protective mechanism not present in males [21]. These findings indicate that sex hormones may play at least a partial role in sex-specific adipose tissue function.

Because sex dimorphism consists of several components, the direct role of hormones is difficult to determine in vivo. Cohorts undergoing gender-affirming treatment could act as a model for such studies. To our knowledge, there is only one previous study (19 transgender women [TW] and 17 transgender men [TM]) that have investigated the effects of gender-affirming treatment in adipose tissue [22]. However, neither the inflammatory status of adipose tissue nor the effects of gonadal downregulation were examined.

We hypothesized that hormone treatment may affect important adipose tissue features and in the current study, we aimed to comprehensively examine the effects of gender-affirming treatment on in vitro adipocyte lipolysis, fat cell size and secretion of 16 cytokines with known impact on adipose tissue metabolism.

## Methods

### Study design and participants

This study is part of a single-center interventional prospective study (GEnder Dysphoria Treatment in Sweden study, GETS, Clinical Trials Identifier NCT02518009) that has been described previously [23, 24]. The study, conducted between April 2015 and May 2021, recruited individuals who had been referred and accepted for gender-affirming hormonal treatment of gender dysphoria at the Andrology Sexual Medicine and Transgender Medicine unit (ANOVA), Karolinska University Hospital, Stockholm, Sweden. All inclusion and exclusion criteria can be found in a previous publication [23]. Inclusion criteria were age 20–40 years, individuals with gender dysphoria who had been accepted for hormonal treatment and who had requested as much hormonal feminization or masculinization as possible and were willing to participate in the study. Exclusion criteria included an already started hormonal therapy, ongoing infectious disease, treatment with warfarin or other anticoagulant, history of cardiovascular disease, type 1 diabetes, serious psychiatric or somatic disease, alcohol or drug abuse, and language difficulties. Those who identified themselves as non-binary and wanted partial hormonal treatment were also excluded from participation. All individuals were initially treated with an injection of a gonadotropin releasing hormone (GnRH) antagonist (degarelix 240 mg subcutaneously). This strategy results in an immediate reduction in gonadotropin secretion and bring sex hormone levels (estradiol and testosterone) to castrate levels within 24 h. In clinical practice, inhibition of endogenous sex hormone production is usually combined with gender-affirming hormonal treatment. However, initial GnRH antagonist treatment provides an opportunity to compare in vivo effects of an environment without the presence of androgens and estrogens in adipose tissue. The subsequent gender-affirming hormone treatment was started at 4 weeks, after post-castration assessments were made. TM were treated with testosterone injections (testosterone undecanoate 1000 mg intramuscularly) with the first two injections given with a 6-week interval and thereafter one injection every 10th week. Dose adjustments were made to maintain androgen levels within the normal adult male reference range. In TW, androgen suppression was maintained with a GnRH analogue (Triptorelin pamoate [Pamorelin®] 11.25 mg administered intramuscularly every third month). Estradiol was administered transdermally (gel or patches), orally, or in a few cases intramuscularly (estrogenpolyphosphate). The estradiol doses given were either 1–2 mg gel applied daily, 100–200 μg/24 h patches, delivered twice weekly, 4–8 mg orally, or 80 mg IM every 2–4 weeks. The different routes of delivery used in the study reflect clinical practice.

The subjects were examined following an overnight fasting at four time points; baseline, 4 weeks after initiated gonadal suppression of endogenous sex hormones but before gender-affirming hormone treatment and 3 and 11 months after initiated gender-affirming hormone treatment. In total, 34 subjects, 17 TW and 17 TM, were initially included in the study. Venous blood samples were obtained. An abdominal subcutaneous fat biopsy in the paraumbilical region was obtained by needle aspiration following local anesthesia with prilocaine 5 mg/ml (without adrenaline). No adipose tissue biopsies were obtained in two TW.

### Fat cell size

Adipocytes were isolated from the adipose tissue biopsy after collagenase treatment [25]. By measuring the diameter of 100 isolated adipocytes using light microscopy, the mean fat cell volume was calculated using a formula as described previously [20].

### Lipolysis

Lipolysis experiments were performed according to a standard protocol [26]. Isolated fat cells from collagenase treated adipose tissue biopsies were incubated for 2 h at 37 °C in a lipolysis buffer (Krebs Ringer Phosphate buffer containing bovine albumin (20 g/l), glucose (1 g/l) and ascorbic acid (0.1 g/l).) in the absence (basal lipolysis) or presence (stimulated lipolysis) of various concentrations of noradrenaline and isoprenaline. At the end of incubation, glycerol release into the medium was determined and related to number of incubated fat cells. During fat mobilization in adipocytes by lipolysis, a triglyceride molecule is broken down into one glycerol molecule and three fatty acid molecules. The release of glycerol from the fat cells is a more accurate measure of lipolysis compared to fatty acid release as some of the fatty acids may be re-esterified in the adipocyte. Glycerol, on the other hand, is not reused in adipocytes as they lack glycerol kinase. As there is no consensus on how to express lipolysis, we used both per lipid weight and per the number of fat cells as well as the ratio between stimulated/basal lipolysis.

### Adipokine secretion and blood sample analyses

The selection of cytokines was based on previous studies where the cytokine had either shown a strong association to adipose tissue metabolism or to be modulated by sex hormones. These included inflammatory factors (interferon gamma (IFN-γ), interleukin (IL) 1 beta (IL-1β), IL-6, chemokine IL-8, macrophage migration inhibitory factor (MIF), tumor necrosis factor alfa (TNF-α), Plasminogen activator inhibitor-1 (PAI-1), retinol-binding protein 4 (RBP4), monocyte chemoattractant protein 1 (MCP-1) and IL-18), anti-inflammatory factors (IL-1 receptor antagonist (IL-1RA), IL-10 and adiponectin) as well as some factors with reported dual functions, including both pro-inflammatory and anti-inflammatory effects depending on the environment (complement component 3a (C3a), IL-2 and IL-17a). Supplementary table S1 depicts some of the studies used for selection but should by no means be regarded as a comprehensive list of all published data on these cytokines.

Explants of subcutaneous white adipose tissue were incubated as described previously [27]. In brief, WAT explants were incubated for 2 h at 37 °C in 1 mL/100 mg tissue in the lipolysis buffer (see above). Fifty µl of the total incubation volume were used to analyze the above-listed 16 cytokines in the adipose tissue of both TM and TW at all 4 time points by Luminex multiplex immunoassay (ProcartaPlex, ThermoFisher, Vienna, Austria) according to manufacturer´s instructions. Data were collected using Mapgix (Luminex xMAP™ Corporation, Austin, TX USA) and expressed as picograms per milliliter (pg/ml). The alignment between the observed and expected concentrations in standard curves, curve fit and the amount of counted beads (>50) were used to validate the assay for individual cytokines. Sensitivity and detection limits of this immunoassay are indicated in Supplementary Table S2.

All standard laboratory analyses on blood samples were performed at the ISO accredited Karolinska University Laboratory, Stockholm, Sweden. Plasma concentrations of total cholesterol, high-density lipoprotein (HDL) cholesterol, and triglycerides were quantified using enzymatic photometric methods. Low-density lipoprotein (LDL) cholesterol was calculated using the Friedewald equation when triglyceride levels were below 4.0 mmol/L. Estradiol and testosterone levels were determined through electrochemiluminescence immunoassays. All analyses were performed on Roche Cobas 8000 or 6000 series analyzers (Roche Diagnostics Scandinavia AB, Solna, Sweden), employing reagents from the same manufacturer. For cases presenting with low testosterone levels, quantification was further refined using liquid chromatography–tandem mass spectrometry (LC–MS/MS), incorporating internal deuterium-labeled isotopic standards for enhanced precision.

### Statistics

Time effects, i.e., comparison of pre- and post-intervention data, were derived using mixed linear models with time as a fixed effect and subject/time as a random effect to account for the repeated measures design. The Linear and Nonlinear Mixed Effects Models (nlme) library in R version 3.5.5 was used for this purpose. All dependent variables were treated as independent hypothesis tests and the correction for multiple hypothesis tests was calculated using the Benjamini–Hochberg false discovery rate, where a false discovery rate of 1% was considered significant. Statistical analysis for secreted cytokines was performed only for individuals without any missing time points and, therefore, two-way ANOVA for repeated measures and multiple comparisons was used.

## Results

Of the 17 TW and 17 TM included in the study, the measurements of anthropometry were available for 15 TW and 17 TM. Clinical characteristics of the individuals undergoing gender-affirming treatment and number of subjects for each datapoint are presented in Table 2. The mean age in the cohort was 26 ± 4 years (TW 26 ± 4 years and TM 25 ± 5 years, respectively). There were no significant changes in body weight, BMI, glucose, insulin or insulin resistance estimated by HOMA-IR in either group during the study period (Table 1). As expected, in both transmen and transwomen, endogenous sex hormone production was downregulated following 1 month of GnRH antagonist treatment (termed T0) and gender-affirming hormone treatment resulted in increased levels of sex hormones (see Table 1). Lipid levels for TW and TM are also shown in Table 2. In TW, there were no change in total cholesterol, TG or LDL (*p* = 0.13–0.98). HDL cholesterol increased after 3 months but returned to baseline after 11 months after gender-affirming treatment was started. In TM, total cholesterol was also unchanged, HDL decreased from 1.6 to 1.2 mmol/L (*p* = 0.0002) 11 months after initiation of gender-affirming treatment and there was a small decrease in TG after 3 months (Table 1).

**Table 1:** Characterization of transwomen and transmen before initiated treatment (T00), after gonadal downregulation (T0), 3 months (T3) and 11 months (T11) after gender-affirming hormonal treatment was started

Time	T00	T0	T3	T11	T00 vs T0	T00 vs T3	T00 vs T11
	Median (IQR)	Median (IQR)	Median (IQR)	Median (IQR)	*p*-value	*p*-value	*p*-value
**Transwomen**							
Age (years)	26 (24–27)						
Weight (kg)	67 (64–80)	65 (63–84)	68 (64–83)	73 (66–85)	0.94	0.38	0.07
Height (cm)	180 (175–183)						
Body mass index (kg/m^2^)	21.7 (19.5–24.7)	20.4 (19.3–25.4)	21.4 (19.9–25.2)	21.9 (20.6–25.4)	0.81	0.48	0.06
Glucose (mmol/L)	5.3 (4.9–5.6)	5.2 (5.1–5.4)	5.1 (4.9–5.3)	5.1 (5.0–5.3)	0.76	0.15	0.64
Insulin (mU/L)	7.6 (6.1–9.6)	6.9 (5.4–11.0)	7.3 (4.6–9.9)	9.4 (5.7–12.0)	0.78	0.86	0.16
HOMA-IR	1.86 (1.52–2.09)	1.56 (1.27–2.35)	1.61 (1.05–2.34)	2.06 (1.37–2.91)	0.76	0.52	0.31
Cholesterol (mmol/L)	3.8 (3.4–4.4)	4.1 (3.5–4.5)	3.7 (3.0–4.7)	3.8 (3.5–4.2)	0.06	0.72	0.41
HDL cholesterol (mmol/L)	1.3 (1.1–1.6)	1.5 (1.2–1.7)	1.4 (1.3–1.9)	1.4 (1.1–1.6)	0.83	0.05	0.98
LDL cholesterol (mmol/L)	2.0 (1.7–2.8)	2.3 (1.7–2.9)	1.6 (1.3–1.6)	2.1 (1.6–2.4)	0.31	0.14	0.13
Triglycerides (mmol/L)	0.7 (0.5–1.0)	0.7 (0.6–1.1)	0.6 (0.5–0.8)	0.7 (0.4–0.9)	0.14	0.81	0.90
Estradiol (pmol/L)	77 (61–110)	16 (10–45)	232 (199–568)	442 (237–575)	0.28	<0.0001	<0.0001
Testosterone (nmol/L)	19 (18–22)	0.5 (0.3–0.6)	0.4 (0.4–0.8)	0.6 (0.4–0.7)	<0.0001	<0.0001	<0.0001
Sex hormone binding globulin (nmol/L)	41 (34–52)		70 (48–84)	57 (42–79)		0.004	0.008
**Transmen**							
Age (years)	24 (22–29)						
Weight (kg)	59 (51–87)	60 (50–83)	65 (54–83)	67 (58–80)	0.67	0.77	0.93
Height (cm)	167 (164–171)						
Body mass index (kg/m^2^)	20.7 (19.6–32.9)	20.7 (18.9–29.5)	22.1 (20.1–29.8)	22.9 (20.7–27.7)	0.54	0.90	0.95
Glucose (mmol/L)	5.1 (4.9–5.3)	5.1 (4.9–5.5)	5.2 (5.0–5.6)	5.3 (5.1–5.5)	1.00	0.18	0.07
Insulin (mU/L)	8.8 (6.3–13.5)	7.6 (5.7–13.5)	8.4 (6.8–10.5)	8.6 (6.4–11)	0.87	0.51	0.65
HOMA-IR	2.00 (1.45–3.23)	1.81 (1.20–3.23)	1.94 (1.55–2.71)	2.09 (1.52–5.34)	0.80	0.62	0.95
Cholesterol (mmol/L)	4.1 (3.6–4.6)	4.3 (4.0–4.6)	4.2 (3.6–4.6)	3.9 (3.3–4.4)	0.10	0.48	0.46
HDL cholesterol (mmol/L)	1.6 (1.3–1.9)	1.8 (1.2–2.1)	1.4 (1.1–1.7)	1.3 (1.0–1.4)	0.14	0.14	0.0002
LDL cholesterol (mmol/L)	1.9 (1.5–2.8)	2.2 (1.8–2.7)	2.2 (2.0–2.7)	2.1 (1.6–2.7)	0.15	0.89	0.98
Triglycerides (mmol/L)	0.8 (0.6–1.0)	0.7 (0.6–0.9)	0.9 (0.6–1.2)	1.0 (0.7–1.2)	0.06	0.10	0.04
Estradiol (pmol/L)	390 (218–603)	24 (13–40)	124 (76–187)	183 (143–248)	<0.0001	0.0002	<0.0001
Testosterone (nmol/L)	1 (0.6–1.4)	0.9 (0.5–1.1)	21 (14–27)	21 (18–29)	0.95	<0.0001	<0.0001
Sex hormone binding globulin (nmol/L)	67 (43–101)		35 (26–45)	24 (19–39)		0.002	0.0005

**Table 2:** Fat cell volume and lipolysis in transwomen and transmen before initiated treatment (T00), after gonadal downregulation (T0), 3 months (T3) and 11 months (T11) after gender-affirming hormonal treatment was started

Time	T00		T0		T3		T11		T00 vs T0	T00 vs T3	T00 vs T12
	*n*	Median (IQR)	*n*	Median (IQR)	*n*	Median (IQR)	*n*	Median (IQR)	*p*-value	*p*-value	*p*-value
**Transwomen**											
Fat cell volume (pL)	15	387 (209–561)	12	347 (271–534)	11	340 (222–424)	12	342 (284–517)	0.80	0.47	0.74
Basal lipolys (µmol glycerol/g TG/2 h)	14	0.70 (0.57–0.88)	12	0.64 (0.54–0.82)	11	0.60 (0.43–0.98)	12	0.88 (0.43–2.10)	0.55	0.75	0.09
Noradrenaline induced lipolysis (µmol glycerol/g TG/2 h)	14	2.67 (1.23–3.64)	12	2.98 (1.39–4.08)	11	2.36 (1.42–4.13)	12	3.00 (2.06–3.95)	0.69	0.90	0.16
Isoprenaline induced lipolysis (µmol glycerol/g TG/2 h)	14	8.46 (3.51–9.40)	12	5.79 (3.47–11.11)	11	8.48 (4.08–9.87)	12	7.16 (6.35–8.85)	0.89	0.85	0.34
Basal lipolysis (µmol glycerol/×10^7^ cells/2 h)	14	2.72 (1.27–3.55)	12	2.03 (1.01–3.09)	11	1.56 (0.96–2.27)	12	3.21 (1.46–7.77)	0.32	0.47	0.19
Noradrenaline stimulated lipolysis (µmol glycerol/×10^7^ cells/2 h)	14	6.20 (4.79–13.10)	12	8.90 (5.23–14.25)	11	5.15 (4.50–13.38)	12	9.26 (6.66–21.94)	0.79	0.86	0.14
Isoprenaline stimulated lipolysis (µmol glycerol/×10^7^ cells/2 h)	14	18.09 (15.24–32.89)	12	19.26 (15.30–29.04)	11	19.33 (14.67–29.84)	12	22.38 (16.46–35.61)	0.76	0.84	0.72
Noradrenaline/Basal lipolysis (ratio)	14	2.91 (1.85–4.41)	12	3.57 (2.27–6.62)	11	3.37 (2.70–5.68)	12	3.47 (2.31–4.59)	0.16	0.63	0.53
Isoprenaline/Basal lipolysis (ratio)	14	11.70 (4.82–14.56)	12	10.22 (6.73–16.62)	11	14.37 (5.66–18.62)	12	7.68 (5.03–15.30)	0.86	0.59	0.71
**Transmen**											
Fat cell volume (pL)	14	430 (299–576)	14	396 (308–519)	14	545 (349–680)	16	442 (300–609)	0.76	0.09	0.87
Basal lipolys (µmol glycerol/g TG/2 h)	14	0.74 (0.34–1.02)	14	0.94 (0.36–1.20)	14	0.96 (0.52–1.30)	16	1.05 (0.79–1.48)	0.39	0.30	0.20
Noradrenaline induced lipolysis (µmol glycerol/g TG/2 h)	14	2.77 (1.54–3.72)	14	3.81 (2.35–5.87)	14	2.72 (1.57–3.44)	16	2.71 (2.04–3.33)	0.04	0.59	0.57
Isoprenaline induced lipolysis (µmol glycerol/g TG/2 h)	14	5.91 (5.14–7.38)	14	7.48 (6.04–8.77)	14	6.27 (5.43–7.95)	16	5.77 (5.09–9.12)	0.06	0.70	0.41
Basal lipolysis (µmol glycerol/×10^7^ cells/2 h)	14	2.35 (1.26–4.55)	14	2.94 (1.86–6.03)	14	4.30 (2.02–7.54)	16	3.09 (2.68–5.51)	0.48	0.05	0.15
Noradrenaline stimulated lipolysis (µmol glycerol/×10^7^ cells/2 h)	14	10.70 (4.68–16.60)	14	14.52 (6.80–24.28)	14	13.74 (6.66–22.35)	16	9.69 (5.93–15.81)	0.07	0.54	0.49
Isoprenaline stimulated lipolysis (µmol glycerol/×10^7^ cells/2 h)	14	23.73 (17.68–27.88)	14	31.12 (18.30–34.94)	14	26.85 (25.15–36.64)	16	23.19 (19.68–32.80)	0.11	0.10	0.42
Noradrenaline/Basal lipolysis (ratio)	14	3.64 (2.38–5.45)	14	4.00 (3.08–7.65)	14	3.25 (1.54–4.97)	16	2.45 (1.63–3.26)	0.10	0.74	0.04
Isoprenaline/Basal lipolysis (ratio)	14	8.90 (5.91–13.01)	14	9.06 (6.09–13.30)	14	7.87 (4.97–13.35)	16	6.19 (4.84–8.60)	0.92	0.95	0.15

*TG* Triglycerides

### Several factors secreted by adipose tissue are affected by hormone washout

**Fig. 1:**
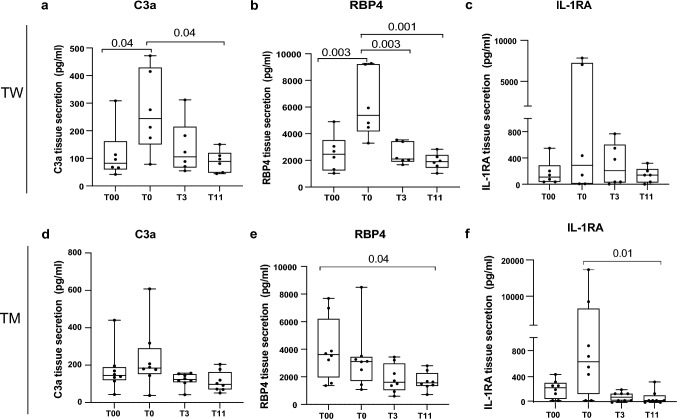
Significantly regulated secreted adipokine. Adipokines C3a (**a**, **d**), RBP4 (**b**, **e**) and IL-1RA (**c**, **f**) were measured by Luminex multiplexing assay in TW (*n* = 6) and TM (*n* = 8). Measurements were performed before initiated treatment (T00), 1 month after initiated gonadal down-regulation (T0), 3 months (T3) and 11 months (T11) after gender-affirming hormonal treatment was started. The secretion is expressed as pg/ml. Differences between the groups were calculated using one-way ANOVA with multiple comparisons

**Fig. 2:**
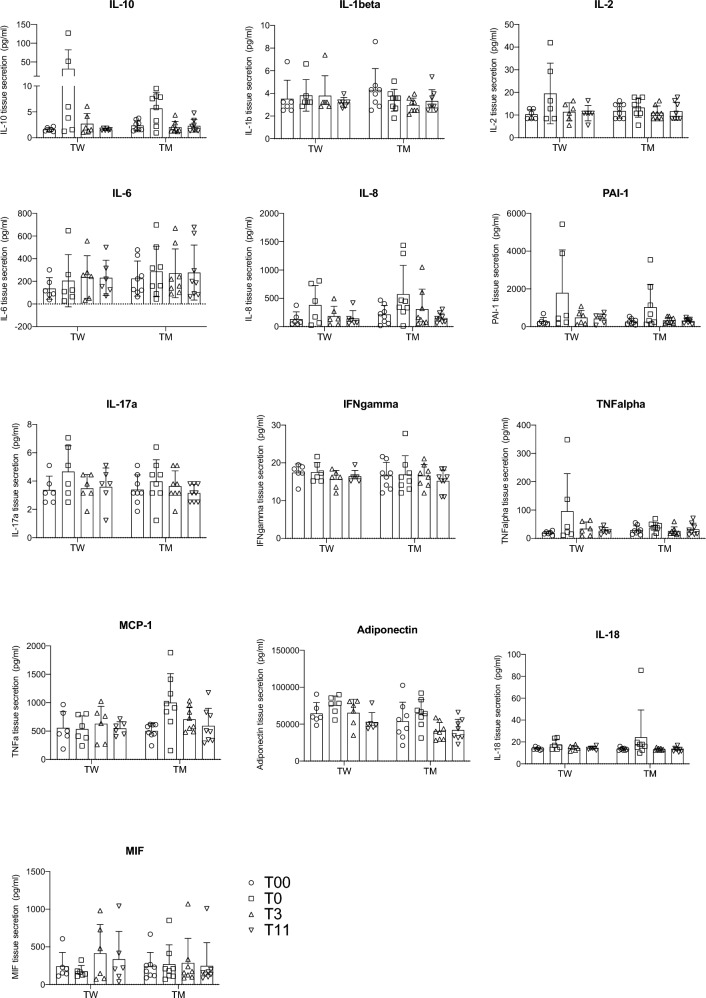
Secretion of the adipokines that are not affected by gender-affirming therapy. Adipokines were measured by Luminex multiplexing assay in TW (*n* = 6) and TM (*n* = 8). Measurements were performed before initiated treatment (T00), 1 month after initiated gonadal down-regulation (T0), 3 months (T3) and 11 months (T11) after gender-affirming hormonal treatment was started. The secretion is expressed as pg/ml. Differences between the groups were calculated using one-way ANOVA with multiple comparisons

Although there were no systemic changes in insulin sensitivity estimated with HOMA-IR in the treated patients, we further investigated if adipose tissue-secreted factors can be locally affected by sex hormone changes during gonadal downregulation and gender-affirming hormone treatment. Of the 16 cytokines that we selected, only 3 were affected by the treatment. A complement factor C3a was significantly increased by the gonadal downregulation in TW and then normalized following 11 months of estrogen treatment (Fig. 1a). A similar upregulation and normalization were observed for retinol binding protein 4 (RBP4) in TW (Fig. 1b), suggesting that removal of testosterone potentiates secretion of both these factors. Although IL-1RA showed a similar trend of regulation as RBP4 and C3a, the change was not significant (Fig. 1c). In TM, no changes were observed for C3a (Fig. 1d), but RBP4 was significantly downregulated by the hormonal intervention (comparing before (T00) and after 11 months (T11) after gender-affirming hormonal treatment was started) (Fig. 1e). Interestingly, IL-1RA was significantly higher after gonadal hormone downregulation in TM compared to after the 11 months treatment. IL-1RA showed a trend for upregulation after hormone washout (one month after removal of estrogens), but this effect was not significant (Fig. 1f). The levels of the thirteen cytokines that did not change significantly during gonadal downregulation or gender-affirming hormone treatment are displayed in Fig. 2.

### No major changes in fat cell size and lipolysis

**Fig. 3:**
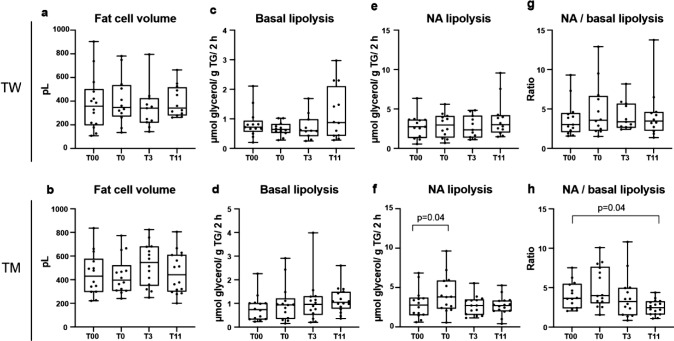
Fat cell volume and lipolysis. Values for **a**, **b** Fat cell volume **c**, **d** Basal lipolysis **e**, **f** Noradrenaline stimulated lipolysis **g**, **h** ratio of Noradrenaline/basal lipolysis for TW (*n* = 14) and TM (*n* = 16) are shown. Measurements were performed before initiated treatment (T00), 1 month after initiated gonadal down-regulation (T0), 3 months (T3) and 11 months (T11) after gender-affirming hormonal treatment was started

We further wanted to confirm whether the previously shown changes in adipocyte cell size and lipolysis following gender-affirming treatment [22] were also seen in our cohort. However, no changes in fat cell size were seen in TM or TW following gonadal downregulation or hormonal treatment in our study (Table 2 and Fig. 3a, b, *p* = 0.06–0.87). In addition, no alterations in basal or stimulated lipolysis were detected in TW (Fig. 3c, e, g) regardless of whether lipolysis was expressed as per gram lipid, per cell unit for basal, noradrenaline-stimulated, isoprenaline-stimulated, or as a ratio of noradrenaline/basal lipolysis (*p* = 0.06–0.99) (Table 3).

TM showed no changes in basal lipolysis (Fig. 3d), but an increase in noradrenaline-stimulated lipolysis following gonadal downregulation that returned to baseline levels at the examination 11 months after baseline (Fig. 3f). The ratio NA/basal lipolysis ratio decreased at 11 months compared to baseline in TM both when expressed as per gram triglycerides or per cell (Fig. 3h, *p* = 0.04). No other alterations in basal or stimulated lipolysis were detected in TM when lipolysis was expressed as per gram lipid, per cell unit for basal or isoprenaline-stimulated lipolysis, (Table 2, *p* = 0.10–0.95).

## Discussion

To our knowledge, this is the first study to examine the effects of gonadal downregulation and gender-affirming sex hormone treatment on adipose tissue cytokine levels, and the second to study the effects on lipolysis in both TW and TM. We report transiently elevated levels of C3a and RBP-4 following gonadal downregulation in TW, which was normalized by estrogen treatment. In TM, two cytokines were altered by the treatment (RBP4 and IL1RA), and a transient increase in NA-stimulated lipolysis was seen. No significant changes in fat cell size was observed in either TW or TM.

The effect of estrogen on lipolysis is controversial. Previous in vitro studies have shown that both estrogen and testosterone treatment resulted in inhibition of stimulated lipolysis [6, 19]. On the other hand, another study showed that estradiol treatment of subcutaneous adipocytes resulted in increased lipolysis [28]. Furthermore, some of the biological effects traditionally attributed to testosterone acting via the androgen receptor may in part be dependent on estradiol produced in the testis or aromatized from testosterone in several tissues, as discussed previously [29]. One might speculate that the transient increase of stimulated lipolysis in TM may be a result of decreased inhibition of stimulated lipolysis by estrogen. However, the fact that this was not observed in TW and data showing that estrogen does not regulate lipolysis directly does not support this hypothesis [30]. These discrepancies between the studies could be due to sampling methods, hormone administration and/or gender-specific adrenergic receptor distribution [31].

A study similar to ours reported an increase in abdominal and gluteal subcutaneous fat cell size in TW, a decrease in abdominal subcutaneous fat cells in TM, a decrease in basal lipolysis in TW and increase in basal lipolysis in TM [22]. It should be noted that both our study and Elbers et al. [22] examined relatively small cohorts where interindividual variation was quite high and treatment protocols were different.

Previous findings regarding insulin sensitivity following gender-affirming hormone treatment are also conflicting; both unchanged [32, 33] (and our study), increased [12] (in TM) and decreased [34] insulin sensitivity have been reported [12]. Furthermore, in a recent large retrospective study, transgender individuals did not display an increased incidence of type 2 diabetes compared with the general population [35]. The changes in lipid profile in our study, i.e., a tendency towards decreased HDL cholesterol and increased triglycerides in TM and transiently increased HDL cholesterol levels in TW, are in line with earlier reports [36, 37]. In addition, the ENIGI (European Network for the Investigation of Gender Incongruence) study, including over 2600 individuals, reported unfavorable lipid changes and increased risk of cardiovascular disease in TM, but decrease of total cholesterol, HDL-c, LDL-c. and triglycerides in TW [38].

The observed increase in RBP4 and C3a secretion caused by a GnRH antagonist in TW could be explained not only by suppression of testosterone but also lower levels of estradiol (in connection to decreased production in the testis and aromatization of testosterone to estradiol in other tissues). One might speculate that estrogen has a negative effect on RBP4 and C3a secretion, which is reversed by GnRH antagonist treatment. However, the decrease of both RBP4 and the anti-inflammatory IL-1RA (and a trend for C3a) by testosterone treatment in TM suggests that testosterone may directly regulate these proteins and provides a novel insight into the potential direct effect of androgens.

While there are previous studies that appear to link these three secreted factors to sex hormones, most of them are cohort-based and do not distinguish between direct and confounding effects. The RBP4 has been found to be higher in non-obese and non-diabetic men compared to women [39] and correlated with higher testosterone levels in women with polycystic ovary syndrome [40], suggesting a positive association between testosterone and RBP4, whereas here we show a negative effect of testosterone treatment. The observed positive correlations reported by previous studies may not be due to hormone levels but rather to a confounding factor related to either sex or the presence of polycystic ovary syndrome. Furthermore, the changes in RBP4 levels reported therein could be adipose tissue-specific and would not necessarily be consistent with serum measurements used in previous studies. Hence, our finding may have implications for future disentangling of the direct effects of sex hormones on RBP4 as well as for distinguishing between local versus systemic effects.

Comparatively little research has been published on the relationship between sex hormones and IL-1RA. A recent study suggested that IL-1RA might protect androgen-dependent cancer cells from inflammatory damage and therefore contribute to prostate cancer progression [41]. The fact that IL-1RA is a potent anti-inflammatory factor, possibly directly regulated by sex hormones, adds a new and unique insight to our findings.

Interestingly, even C3a regulation by sex hormones can be debated. A 2016 systematic review of 13 studies reported an increase in C3 complement levels in menopausal women receiving hormone replacement therapy [42]. As the authors of the review note, subjects in the studies were not well matched for demographic or health characteristics, making the association less reliable. Another recent study demonstrated that estradiol treatment of rats subjected to brain death procedures lowers C3 levels [43]. Although the latter model is vastly different and include confounding factors such as brain death, it is in line with our findings that estradiol lowers levels of C3 in adipose tissue.

A strength of the current study is the longitudinal design with four different time points that help to distinguish between possible effects of endogenous sex hormone downregulation versus gender-affirming hormone treatment. The translational approach of the present study with both clinical data and investigations of cellular effects in human adipose tissue is another strength.

A limitation of the present study, as with the few previous translational human studies of gender dysphoria, is the relatively small number of subjects and a rather short follow-up period, which makes it difficult to find small effects due to inter-individual variation, and impossible to evaluate long-term effects on possible changes in adipose tissue expansion and metabolism. Therefore, we cannot exclude that there are small effects of gonadal downregulation or gender-affirming hormone treatment on cytokine secretion or lipolysis. Both cytokine secretion profiles and lipolysis in adipose tissue are well known to be dependent on BMI. In this study, the majority of individuals were lean and we did not have enough power to analyze lean and overweight/obese individuals separately. Another limitation is that we only investigated subcutaneous adipose tissue. It is known that adipose tissue function differs when comparing fat from the abdominal subcutaneous, gluteal subcutaneous, or visceral depot [13, 44].

In conclusion, the current study indicate that gender-affirming hormonal treatment does not result in major alterations in the metabolic profile in adipose tissue regarding lipolysis or cytokine production. This could be an indication for clinicians that, at least in the short-term, gender-affirming hormone treatment is safe in terms of metabolic risk in adipose tissue metabolism.

### Supplementary Information

Below is the link to the electronic supplementary material.

Supplementary file1 (DOCX 91 KB)

## Data Availability

Data are available from the corresponding author for any interested researcher who meets the criteria for access to confidential data and upon reasonable request.
