# Supplementary material for: Adipokine secretion and lipolysis following gender-affirming treatment in transgender individuals
Source: J Endocrinol Invest. 2024 Mar 9;47(9):2249–60. doi: 10.1007/s40618-024-02323-4 (PMC11368987; doi:10.1007/s40618-024-02323-4)
Supplement: Supplementary file 1 — Supplementary file1 (DOCX 91 KB) [file 40618_2024_2323_MOESM1_ESM.docx]

| **ProcartaPlex Mix & Match Human 16-plex ( Mapgix)** | | | |
| --- | --- | --- | --- |
| **Number** | **Target name** | **Bead region** | **Standard concentration range (pg/ml)** |
| 1 | C3a | 14 | 141600 – 34.5 |
| 2 | IL-1b | 18 | 10400 – 2.53 |
| 3 | IL-2 | 19 | 29100 – 7.1 |
| 4 | IL-6 | 25 | 33000 – 8.05 |
| 5 | IL-8 | 27 | 8950 – 2.18 |
| 6 | IL-10 | 28 | 6150 – 1.5 |
| 7 | RBP4 | 33 | 800000 – 195.3 |
| 8 | PAI-1 | 35 | 105100 – 24.7 |
| 9 | IL-17a | 36 | 7650 – 1.86 |
| 10 | IL-1RA | 38 | 143400 – 35 |
| 11 | IFN-Ɣ | 43 | 48900 – 12.15 |
| 12 | TNF-⍺ | 45 | 26000 – 6.34 |
| 13 | MCP-1 | 51 | 15400 – 3.75 |
| 14 | Adiponectin | 57 | 640900 – 156.4 |
| 15 | IL-18 | 66 | 58000 – 14.1 |
| 16 | MIF | 67 | 850 – 0.2 |

**Supplementary table S1.**

The detection ranges and bead regions of Luminex multiplexing assay (for tissue explants).

**Supplementary table S2. List of analyzed cytokines/secreted factor in adipose tissue with references to previous studies.**

*****anti-inflammatory factors

| **Secreted factor** | **Function in adipose tissue** | | **Reported sex differences/regulation by hormones** |
| --- | --- | --- | --- |
|  | Inflammation | Metabolism |  |
| TNF alfa | (1) | (2) | (3, 4) |
| IL-1 beta | (5) | (6) | (4, 7, 8) |
| PAI-1 | (9) | (10) | (11, 12) |
| RBP4 | (13) | (14) | (15, 16) |
| C3a | (17) | (18) | (19) |
| MIF | (20, 21) | (22, 23) | (24) |
| IL18 | (25) | (25, 26) | (27) |
| MCP1 | (28, 29) | (28, 29) | (30) |
| IFNg | (31) | (32) | (33, 34) |
| IL1RA* | (35) | (36) | (37) |
| IL17 | (38) | (39) | (40) |
| IL8 | (41) | (42) | (27, 43) |
| IL6 | (44) | (45) | (3, 4, 46) |
| IL2 | (47) | (48) | (49) |
| Adiponectin* | (50) | (51) | (52) |
| IL-10* | (53) | (54) | (8, 55) |

1. Shi C, Zhu L, Chen X, Gu N, Chen L, Zhu L, et al. IL-6 and TNF-alpha induced obesity-related inflammatory response through transcriptional regulation of miR-146b. J Interferon Cytokine Res. 2014;34(5):342-8.

2. Du X, Liu M, Tai W, Yu H, Hao X, Loor JJ, et al. Tumor necrosis factor-alpha promotes lipolysis and reduces insulin sensitivity by activating nuclear factor kappa B and c-Jun N-terminal kinase in primary bovine adipocytes. J Dairy Sci. 2022;105(10):8426-38.

3. Iannantuoni F, Salazar JD, Martinez de Maranon A, Banuls C, Lopez-Domenech S, Rocha M, et al. Testosterone administration increases leukocyte-endothelium interactions and inflammation in transgender men. Fertil Steril. 2021;115(2):483-9.

4. Bernardi S, Toffoli B, Tonon F, Francica M, Campagnolo E, Ferretti T, et al. Sex Differences in Proatherogenic Cytokine Levels. Int J Mol Sci. 2020;21(11).

5. Nov O, Shapiro H, Ovadia H, Tarnovscki T, Dvir I, Shemesh E, et al. Interleukin-1beta regulates fat-liver crosstalk in obesity by auto-paracrine modulation of adipose tissue inflammation and expandability. PloS one. 2013;8(1):e53626.

6. Bing C. Is interleukin-1beta a culprit in macrophage-adipocyte crosstalk in obesity? Adipocyte. 2015;4(2):149-52.

7. Giltay EJ, Fonk JC, von Blomberg BM, Drexhage HA, Schalkwijk C, Gooren LJ. In vivo effects of sex steroids on lymphocyte responsiveness and immunoglobulin levels in humans. J Clin Endocrinol Metab. 2000;85(4):1648-57.

8. Malkin CJ, Pugh PJ, Jones RD, Kapoor D, Channer KS, Jones TH. The effect of testosterone replacement on endogenous inflammatory cytokines and lipid profiles in hypogonadal men. J Clin Endocrinol Metab. 2004;89(7):3313-8.

9. Wang L, Chen L, Liu Z, Liu Y, Luo M, Chen N, et al. PAI-1 Exacerbates White Adipose Tissue Dysfunction and Metabolic Dysregulation in High Fat Diet-Induced Obesity. Front Pharmacol. 2018;9:1087.

10. Alessi MC, Poggi M, Juhan-Vague I. Plasminogen activator inhibitor-1, adipose tissue and insulin resistance. Current opinion in lipidology. 2007;18(3):240-5.

11. Giltay EJ, Elbers JM, Gooren LJ, Emeis JJ, Kooistra T, Asscheman H, et al. Visceral fat accumulation is an important determinant of PAI-1 levels in young, nonobese men and women: modulation by cross-sex hormone administration. Arterioscler Thromb Vasc Biol. 1998;18(11):1716-22.

12. Asselbergs FW, Williams SM, Hebert PR, Coffey CS, Hillege HL, Navis G, et al. Gender-specific correlations of plasminogen activator inhibitor-1 and tissue plasminogen activator levels with cardiovascular disease-related traits. J Thromb Haemost. 2007;5(2):313-20.

13. Farjo KM, Farjo RA, Halsey S, Moiseyev G, Ma JX. Retinol-binding protein 4 induces inflammation in human endothelial cells by an NADPH oxidase- and nuclear factor kappa B-dependent and retinol-independent mechanism. Mol Cell Biol. 2012;32(24):5103-15.

14. Kilicarslan M, de Weijer BA, Simonyte Sjodin K, Aryal P, Ter Horst KW, Cakir H, et al. RBP4 increases lipolysis in human adipocytes and is associated with increased lipolysis and hepatic insulin resistance in obese women. FASEB J. 2020;34(5):6099-110.

15. Bakshi S, Schmidt HM, Baskin AE, Croniger CM, Thompson CL, Bonfield T, et al. Sexual dimorphism in developmental and diet-dependent circulating retinol binding protein 4. Obes Sci Pract. 2018;4(6):526-34.

16. Wang H, Zhou P, Zou D, Liu Y, Lu X, Liu Z. The role of retinol-binding protein 4 and its relationship with sex hormones in coronary artery disease. Biochem Biophys Res Commun. 2018;506(1):204-10.

17. Shim K, Begum R, Yang C, Wang H. Complement activation in obesity, insulin resistance, and type 2 diabetes mellitus. World J Diabetes. 2020;11(1):1-12.

18. Barbu A, Hamad OA, Lind L, Ekdahl KN, Nilsson B. The role of complement factor C3 in lipid metabolism. Mol Immunol. 2015;67(1):101-7.

19. Gaya da Costa M, Poppelaars F, van Kooten C, Mollnes TE, Tedesco F, Wurzner R, et al. Age and Sex-Associated Changes of Complement Activity and Complement Levels in a Healthy Caucasian Population. Front Immunol. 2018;9:2664.

20. Kim BS, Tilstam PV, Arnke K, Leng L, Ruhl T, Piecychna M, et al. Differential regulation of macrophage activation by the MIF cytokine superfamily members MIF and MIF-2 in adipose tissue during endotoxemia. FASEB J. 2020;34(3):4219-33.

21. Kim BS, Pallua N, Bernhagen J, Bucala R. The macrophage migration inhibitory factor protein superfamily in obesity and wound repair. Experimental & molecular medicine. 2015;47(5):e161.

22. Nishihira J, Sakaue S. Overview of Macrophage Migration Inhibitory Factor (MIF) as a Potential Biomarker Relevant to Adiposity. J Tradit Complement Med. 2012;2(3):186-91.

23. Gligorovska L, Bursac B, Kovacevic S, Velickovic N, Matic G, Djordjevic A. Mif deficiency promotes adiposity in fructose-fed mice. J Endocrinol. 2019;240(2):133-45.

24. Aloisi AM, Pari G, Ceccarelli I, Vecchi I, Ietta F, Lodi L, et al. Gender-related effects of chronic non-malignant pain and opioid therapy on plasma levels of macrophage migration inhibitory factor (MIF). Pain. 2005;115(1-2):142-51.

25. Ahmad R, Thomas R, Kochumon S, Sindhu S. Increased adipose tissue expression of IL-18R and its ligand IL-18 associates with inflammation and insulin resistance in obesity. Immun Inflamm Dis. 2017;5(3):318-35.

26. Troseid M, Seljeflot I, Arnesen H. The role of interleukin-18 in the metabolic syndrome. Cardiovasc Diabetol. 2010;9:11.

27. Takahashi T, Wong P, Ellingson MK, Lucas C, Klein J, Israelow B, et al. Sex differences in immune responses to SARS-CoV-2 that underlie disease outcomes. medRxiv. 2020.

28. Cranford TL, Enos RT, Velazquez KT, McClellan JL, Davis JM, Singh UP, et al. Role of MCP-1 on inflammatory processes and metabolic dysfunction following high-fat feedings in the FVB/N strain. International journal of obesity (2005). 2016;40(5):844-51.

29. Kanda H, Tateya S, Tamori Y, Kotani K, Hiasa K, Kitazawa R, et al. MCP-1 contributes to macrophage infiltration into adipose tissue, insulin resistance, and hepatic steatosis in obesity. The Journal of clinical investigation. 2006;116(6):1494-505.

30. Varghese M, Clemente J, Lerner A, Abrishami S, Islam M, Subbaiah P, et al. Monocyte Trafficking and Polarization Contribute to Sex Differences in Meta-Inflammation. Front Endocrinol (Lausanne). 2022;13:826320.

31. Zhang H, Potter BJ, Cao JM, Zhang C. Interferon-gamma induced adipose tissue inflammation is linked to endothelial dysfunction in type 2 diabetic mice. Basic Res Cardiol. 2011;106(6):1135-45.

32. Huang LY, Chiu CJ, Hsing CH, Hsu YH. Interferon Family Cytokines in Obesity and Insulin Sensitivity. Cells. 2022;11(24).

33. Ono S, Tsujimoto H, Hiraki S, Takahata R, Kinoshita M, Mochizuki H. Sex differences in cytokine production and surface antigen expression of peripheral blood mononuclear cells after surgery. Am J Surg. 2005;190(3):439-44.

34. Pujantell M, Altfeld M. Consequences of sex differences in Type I IFN responses for the regulation of antiviral immunity. Front Immunol. 2022;13:986840.

35. Ballak DB, Stienstra R, Tack CJ, Dinarello CA, van Diepen JA. IL-1 family members in the pathogenesis and treatment of metabolic disease: Focus on adipose tissue inflammation and insulin resistance. Cytokine. 2015;75(2):280-90.

36. Matsuki T, Horai R, Sudo K, Iwakura Y. IL-1 plays an important role in lipid metabolism by regulating insulin levels under physiological conditions. J Exp Med. 2003;198(6):877-88.

37. Furman D, Hejblum BP, Simon N, Jojic V, Dekker CL, Thiebaut R, et al. Systems analysis of sex differences reveals an immunosuppressive role for testosterone in the response to influenza vaccination. Proceedings of the National Academy of Sciences of the United States of America. 2014;111(2):869-74.

38. Chehimi M, Vidal H, Eljaafari A. Pathogenic Role of IL-17-Producing Immune Cells in Obesity, and Related Inflammatory Diseases. J Clin Med. 2017;6(7).

39. Zuniga LA, Shen WJ, Joyce-Shaikh B, Pyatnova EA, Richards AG, Thom C, et al. IL-17 regulates adipogenesis, glucose homeostasis, and obesity. J Immunol. 2010;185(11):6947-59.

40. Zychlinsky Scharff A, Rousseau M, Lacerda Mariano L, Canton T, Consiglio CR, Albert ML, et al. Sex differences in IL-17 contribute to chronicity in male versus female urinary tract infection. JCI Insight. 2019;5(13).

41. Zagotta I, Dimova EY, Debatin KM, Wabitsch M, Kietzmann T, Fischer-Posovszky P. Obesity and inflammation: reduced cytokine expression due to resveratrol in a human in vitro model of inflamed adipose tissue. Front Pharmacol. 2015;6:79.

42. Kobashi C, Asamizu S, Ishiki M, Iwata M, Usui I, Yamazaki K, et al. Inhibitory effect of IL-8 on insulin action in human adipocytes via MAP kinase pathway. J Inflamm (Lond). 2009;6:25.

43. Maggio M, Blackford A, Taub D, Carducci M, Ble A, Metter EJ, et al. Circulating inflammatory cytokine expression in men with prostate cancer undergoing androgen deprivation therapy. J Androl. 2006;27(6):725-8.

44. Han MS, White A, Perry RJ, Camporez JP, Hidalgo J, Shulman GI, et al. Regulation of adipose tissue inflammation by interleukin 6. Proceedings of the National Academy of Sciences of the United States of America. 2020;117(6):2751-60.

45. Wueest S, Konrad D. The role of adipocyte-specific IL-6-type cytokine signaling in FFA and leptin release. Adipocyte. 2018;7(3):226-8.

46. Wilson R, Jenkins C, Miller H, Carr S. The effect of oestrogen on cytokine and antioxidant levels in male to female transsexual patients. Maturitas. 2006;55(1):14-8.

47. Liu R, Nikolajczyk BS. Tissue Immune Cells Fuel Obesity-Associated Inflammation in Adipose Tissue and Beyond. Front Immunol. 2019;10:1587.

48. Kochumon S, Al Madhoun A, Al-Rashed F, Thomas R, Sindhu S, Al-Ozairi E, et al. Elevated adipose tissue associated IL-2 expression in obesity correlates with metabolic inflammation and insulin resistance. Sci Rep. 2020;10(1):16364.

49. Bouman A, Schipper M, Heineman MJ, Faas MM. Gender difference in the non-specific and specific immune response in humans. Am J Reprod Immunol. 2004;52(1):19-26.

50. Kwon H, Pessin JE. Adipokines mediate inflammation and insulin resistance. Front Endocrinol (Lausanne). 2013;4:71.

51. Yanai H, Yoshida H. Beneficial Effects of Adiponectin on Glucose and Lipid Metabolism and Atherosclerotic Progression: Mechanisms and Perspectives. Int J Mol Sci. 2019;20(5).

52. Boyne MS, Bennett NR, Cooper RS, Royal-Thomas TY, Bennett FI, Luke A, et al. Sex-differences in adiponectin levels and body fat distribution: longitudinal observations in Afro-Jamaicans. Diabetes Res Clin Pract. 2010;90(2):e33-6.

53. Acosta JR, Tavira B, Laurencikiene J. Human-specific function of IL-10 in adipose tissue and association to insulin resistance Figshare. 2019.

54. Rajbhandari P, Thomas BJ, Feng AC, Hong C, Wang J, Vergnes L, et al. IL-10 Signaling Remodels Adipose Chromatin Architecture to Limit Thermogenesis and Energy Expenditure. Cell. 2018;172(1-2):218-33 e17.

55. Subramanian N, Tavira B, Hofwimmer K, Gutsmann B, Massier L, Abildgaard J, et al. Sex-specific regulation of IL-10 production in human adipose tissue in obesity. Front Endocrinol (Lausanne). 2022;13:996954.
